# High Anodic-Voltage Focusing of Charge Carriers in Silicon Enables the Etching of Regularly-Arranged Submicrometer Pores at High Density and High Aspect-Ratio

**DOI:** 10.3389/fchem.2018.00582

**Published:** 2018-11-30

**Authors:** Chiara Cozzi, Giovanni Polito, Lucanos M. Strambini, Giuseppe Barillaro

**Affiliations:** ^1^Dipartimento di Ingegneria dell'Informazione, Università di Pisa, Pisa, Italy; ^2^Istituto di Elettronica e di Ingegneria dell'Informazione e delle Telecomunicazioni, Consiglio Nazionale delle Ricerche, Pisa, Italy

**Keywords:** nanostructuring, anodization, porous silicon, submicrometer pores, carrier focusing, high voltage

## Abstract

The anodic dissolution of silicon in acidic electrolytes is a well-known technology enabling the silicon machining to be accurately controlled down to the micrometer scale in low-doped *n*-type silicon electrodes. Attempts to scale down this technology to the submicrometer scale has shown to be challenging, though it premises to enable the fabrication of meso and nano structures/systems that would greatly impact the fields of biosensors and nanomedicine. In this work, we report on the electrochemical etching at high anodic voltages (up to 40 V) of two-dimensional regular arrays of millions pores per square centimeter (up to 30 × 10^6^ cm^−2^) with sub-micrometric diameter (down to ~860 nm), high depth (up to ~40 μm), and high aspect-ratio (up to ~45) using low-doped *n*-type silicon electrodes (resistivity 3–8 Ω cm). The use of high anodic voltages, which are over one order of magnitude higher than that commonly used in electrochemical etching of silicon, tremendously improves hole focusing at the pore tips during the etching and enables, in turn, the control of electrochemical etching of submicrometer-sized pores when spatial period reduces below 2 μm. A theoretical model allows experimental results to be interpreted in terms of an electric-field-enhanced focusing of holes at the tip apex of the pores at high anodic voltages, with respect to the pore base, which leads to a smaller curvature radius of the tip apex and enables, in turn, the etching of pore tips to be preferentially sustained over time and space.

## Introduction

From first discovery to modern days, the possibility of controlling the electrochemical preparation of pores in silicon at nano to micro scales in terms of both size and pattern has fascinated scientists for more than 50 years (Uhlir, 1956).

In 1990, Lehmann and Föll reported for the first time on how to control the anodic dissolution of *n*-type silicon electrodes in low concentration HF-based aqueous electrolytes by back-side illumination, so enabling the etching of regular two-dimensional lattices of pores at the micrometer scale (Lehmann and Föll, 1990). Once a lattice of pits is pre-defined on the silicon surface, size, position, and morphology of pores etched by back-side illumination electrochemical etching (BIEE) on low-doped (i.e., a few Ω cm) *n*-type silicon electrodes was shown to be finely controlled by tuning the etching parameters, such as for instance, doping concentration of silicon, electrolyte composition and temperature, anodic voltage, and current density (Lehmann, 1993; Föll et al., 2002; Matthias et al., 2005; Barillaro and Strambini, 2010). A decade later, Barillaro et al. reported on how to broaden BIEE of silicon to fabricate lattices of linear trenches (Barillaro et al., 2002b), so enabling the fabrication of a multitude of microstructures besides pores (Barillaro et al., 2002a, 2005). In 2012, Barillaro et al. further pushed the BIEE of silicon ahead, showing how to control anodic dissolution anisotropy/isotropy in real-time to enable the fabrication of free-standing microstructures (Polito et al., 2013) and complex microsystems (Bassu et al., 2012) at the micrometer scale. Nowadays, the BIEE of silicon has evolved into a highly versatile microstructuring technology, namely electrochemical micromachining (ECM), with unique features, among which there are high aspect-ratio of etched structures (>100), low roughness of etched surfaces (<10 nm), high etching rate of high aspect-ratio structures (up to 10 μm min^−1^) (Cozzi et al., 2017). As a matter of fact, applications of ECM encompass today a broad range of research fields, from microelectronics (Kemell et al., 2007) and photonics (Surdo et al., 2013), to (bio)sensing (Surdo et al., 2012, 2014) and (nano)medicine (Harding et al., 2016; Delalat et al., 2018).

In spite of the significant technological advancement that has been achieved over the last two decades, the controlled etching of pores with either very large (i.e., >20 μm) or very small (i.e., <1 μm) diameter/spacing has not been achieved yet with BIEE on low-doped *n*-type silicon (Barillaro, 2015). Specifically, maximum diameter and spacing of lattice of pores was shown to have an upper boundary value of about 10 and 20 μm, respectively; on the other hand, minimum size and spacing was reported to be restricted to about 1 and 2 μm, respectively. Both upper and lower boundary values can be ascribed to a poor focusing of photogenerated charge carriers at the pore tips, as both diameter and spacing of pores increase/decrease above/below these values. Moreover, using an anodization voltage value of about 1 V, it was shown that a minimum current density value exists, for a given spacing, setting the minimum diameter value above which the etching of pore lattice can be finely controlled, at least for low-doped *n*-type silicon electrodes (Barillaro and Strambini, 2010).

In this work, the controlled etching of regular arrays of pores with submicrometric diameter (<1 μm) was successfully addressed by back-side illumination electrochemical etching of silicon electrodes with low-resistivity (3–8 Ω cm), using high anodization voltage values (up to 40 V) to effectively focus charge carriers to the pore tips when the spacing of pores is below 2 μm. A theoretical model allowed experimental results to be interpreted in terms of an electric-field enhanced focusing of holes at the tip apex of the pores at high anodic voltages, with respect to the pore base, when pore spacing is smaller than the depletion region width in the silicon electrode. Although the use of high anodization voltages for the electrochemical etching of regular lattices of pores in silicon was already reported (Rönnebeck et al., 1999; Cozzi et al., 2015), this work represents the first report on the controlled etching of sub-micrometric pores with high-density and high aspect-ratio.

## Materials and methods

### Materials and chemicals

CZ-grown *n*-type (100)-oriented silicon wafers with a resistivity of 3–8 Ω cm and covered with a 298-nm-thick silicon dioxide layer, either flat (i.e., without any pattern) or patterned with a two-dimensional (2D) lattice (1 × 1 cm^2^) of square holes with side of 1 μm and spacing (s) of 1.8 μm (hole density ~30 × 10^6^ cm^−2^), were provided by STMicroelectronics (Milan, Italy). Acetone 99 wt%, pentane 99 wt%, 2-propanol 99.8 wt%, and hydrofluoric acid (HF) 48 wt% were purchased from Sigma-Aldrich. Ethanol 99.8 wt% and potassium hydroxide (KOH), pure powder at 85%, were purchased from Fluka Analytical. Sodium lauryl sulfate (SLS) powder was purchased from Carlo Erba Reagents.

### Electrochemical characterization of *n*-type silicon electrode in aqueous HF-based electrolyte by linear sweep voltammetry

A thorough electrochemical characterization (>3 replicates) of flat (i.e., not patterned) *n*-type silicon electrodes in contact with an aqueous HF-based electrolyte (5 vol% HF:95 vol% H_2_O, with 1,000 ppm of Sodium Lauryl Sulfate—SLS—as wetting agent) was carried out by linear sweep voltammetry. Flat silicon electrodes were achieved by cutting the flat silicon wafers in slabs of 2 × 2 cm^2^ and removing the silicon dioxide layer using a solution of HF:ethanol (1:1 by vol.) for 60 s at room temperature. Flat silicon electrodes were loaded in a three-electrodes electrochemical cell containing the HF-based electrolyte (details are provided in the Supplementary Material), and the current flowing through the etch-cell was monitored upon application of voltages varying from 2 to −1.5 V with a sweep rate of −0.1 V s^−1^, under back-side illumination (halogen lamp, 250W) of silicon. Experimental current density-voltage (J-V) curves (Figure S1, Supplementary Material) highlight the presence of an electropolishing current density peak *J*_*peak*_ = 78 ± 3 mA cm^−2^, occurring at a voltage *V*_*peak*_ = 1.2 V, in good agreement with the literature (Lehmann and Föll, 1990; Barillaro and Strambini, 2010).

### Potentiostatic etching of regular arrays of pores at high anodic voltage in aqueous HF-based electrolyte

Pre-patterned *n*-type silicon electrodes were achieved starting from square silicon slabs (2 × 2 cm^2^) cut from the patterned silicon wafers. The pattern was transferred onto the silicon surface by KOH etching, which yielded an array of inverted pyramid-shaped pits. The KOH etching was performed at 50°C for 750 s with a 20 wt% solution of KOH in deionized water, using the patterned silicon dioxide as a masking material. 2-Propanol was added to the KOH solution to increase wetting capability and to improve, in turn, etching uniformity. The silicon dioxide layer was then dissolved using a solution of HF:ethanol (1:1 by vol.) for 60 s at room temperature.

The pre-patterned silicon electrodes were loaded in a three-electrodes electrochemical cell (details are provided in Supplementary Material) containing a HF-based electrolyte (5 vol% HF:95 vol% H_2_O, with 1000 ppm of SLS as wetting agent), and etched for 2,000 s at different anodic voltage values, under back-side illumination of silicon.

The photogenerated etching current density (*J*_*etch*_) was set to a given initial value *J*_*etch*0_ and linearly decreased over time to maintain the pore diameter constant with depth. Three different *J*_*etch*0_ values were tested, namely 13.4, 16.8, and 20.2 mA cm^−2^, which were linearly reduced over time with a rate (α) of −0.938, −1.219, and −1.453 μA s^−1^cm^−2^, respectively, by decreasing the back-side illumination intensity through a reduction of the lamp power (Bassu et al., 2012). The different *J*_*etch*0_ values correspond to different expected porosity (i.e., dissolved silicon to total silicon volumetric ratio) values *P*_*e*_:

(1) $$\begin{array}{r} P_{e}=\frac{J_{etch0}}{J_{peak}} \end{array}$$

namely, 17.2, 21.5, and 25.8%, and, in turn, to different expected pore diameters *d*_*e*_:

(2) $$\begin{array}{r} d_{e}=\sqrt{\;P_{e}\frac{4A_{C}}{\pi }} \end{array}$$

namely, 0.842, 0.942, and 1.032 μm (Table S1, Supplementary Material), being *A*_*C*_ = 1.8 × 1.8 μm^2^ the unit cell area. For each *J*_*etch*0_ value, six different anodization voltage values (*V*_*etch*_) were investigated, namely 1.2, 15, 20, 25, 35, and 40 V. The anodization voltage value is kept constant throughout the whole etching time. For *V*_*etch*_ = 40 V a further current density value *J*_*etch*0_ = 10.07 mA cm^−2^ (*P*_*e*_ = 12.8%, *d*_*e*_ = 0.644 μm) was investigated, with *J*_*etch*0_ linearly reduced over time with α = −0.710 μAs^−1^cm^−2^. Each experiment was replicated at least 3 times for each given *J*_*etch*0_–*V*_*etch*_ pair.

After etching, pre-patterned silicon slabs underwent an overnight static bath in a HF:ethanol (1:4 by vol.) solution to fully remove SLS. A static rinse in ethanol and pentane, respectively, for 300 s followed by drying at 100°C on a hot plate was eventually performed.

All the etched slabs were then diced to allow morphological investigation of the longitudinal cross-section of the pores in the array to be carried out.

### Morphological characterization of regular arrays of pores by SEM microscopy

The morphological characterization of regular arrays of pores resulting from the electrochemical etching of pre-patterned silicon electrodes, at different anodic voltages and current densities, was performed by scanning electron microscope (SEM) using a JEOL JSM-6390. Top-view and cross-section images at different magnifications were acquired at an acceleration voltage of 3 kV. Five different cross-section images were acquired per each slab at both 2,000 × and 5,000 × magnifications, from which pore depth and diameter were experimentally measured. The cross-section images were exploited to perform a statistical analysis using a home-made software routine implemented using Matlab (Mathworks, Inc.), so as to obtain average value and standard deviation of both depth (*h*_*m*_) and diameter (*d*_*m*_) of the etched pores.

Experimental porosity *P*_*m*_ values were obtained from *h*_*m*_ and *d*_*m*_ values through the use of Equation (3), as the ratio between pore cross-sectional area (i.e., A_CS_) and the unit cell area *A*_*C*_, assuming all pores featuring circular cross-section and constant diameter over depth:

(3) $$\begin{array}{r} P_{m}=\frac{A_{CS}}{A_{C}}=\frac{{\pi \left(\frac{d_{m}}{2}\right)}^{2}}{A_{C}} \end{array}$$

Experimental data on pore geometrical features (i.e., *h*_*m*_, *d*_*m*_) were also used to analytically evaluate the total silicon volume dissolved during each etching experiment (*M*_*m*_). In particular, experimental *M*_*m*_ values were evaluated through the use of Equation (4), as the number of pores involved during the etching ρ_*P*_ (i.e., ratio between the etching area *E*_*A*_ of ~0.64 cm^2^ and the unit cell area *A*_*C*_) times the average pore volume (*M*_*P*_):

(4) $$\begin{array}{r} M_{m}=\rho_{P}M_{P}=\frac{E_{A}}{A_{C}}{\pi \left(\frac{d_{m}}{2}\right)}^{2}h_{m} \end{array}$$

The experimental values obtained for the main pore parameters, i.e., diameter (*d*_*m*_), depth (*h*_*m*_), porosity (*P*_*m*_), and volume (*M*_*m*_), resulting from the above described morphological analysis of the SEM images, were compared with the expected values, i.e., *d*_*e*_*, h*_*e*_*, P*_*e*_, and *M*_*e*_, respectively, obtained from the etching parameters, i.e., etching time (*t*_*etch*_) and etching current density (*J*_*etch*_), on the basis of the model proposed by Barillaro and Pieri (2005). In particular, expected diameter (*d*_*e*_) and porosity (*P*_*e*_) were already reported in section Potentiostatic Etching of Regular Arrays of Pores at High Anodic Voltage in Aqueous HF-Based Electrolyte (Equations 1, 2). The expected silicon volume *M*_*e*_ dissolved for any *J*_*etch*0_ value was evaluated through the use of Equation (5), as the ratio of the total charge *Q* supplied during the whole etching experiment $\left(\text{Q}=E_{A}\int_{0}^{t_{etch}}J_{etch}dt\right)$ and the product of silicon dissolution valence *n*_*v*_ = 2.66 (i.e., number of charges needed to dissolve a single silicon atom) (Barillaro and Pieri, 2005), elementary charge *e* (i.e., *e* = 1.602 × 10^−19^ C), and atomic density of silicon *N*_Si_ (i.e., *N*_Si_ = 5 × 10^22^ cm^−3^):

(5) $$\begin{array}{r} M_{e}=\frac{Q}{n_{v}eN_{Si}}=\frac{E_{A}\int_{0}^{t_{etch}}J_{etch}dt}{n_{v}eN_{Si}} \end{array}$$

Eventually, the expected pore depth (*h*_*e*_) was evaluated using *M*_*e*_ and *d*_*e*_ values according to Equation (6):

(6) $$\begin{array}{r} h_{e}=\frac{M_{e}}{P_{e}E_{A}} \end{array}$$

## Results and discussion

### High anodic-voltage controlled etching of regular arrays of high-density high-aspect-ratio pores in *n*-type silicon with diameter down to the sub-micrometric scale

**Figure 1:**
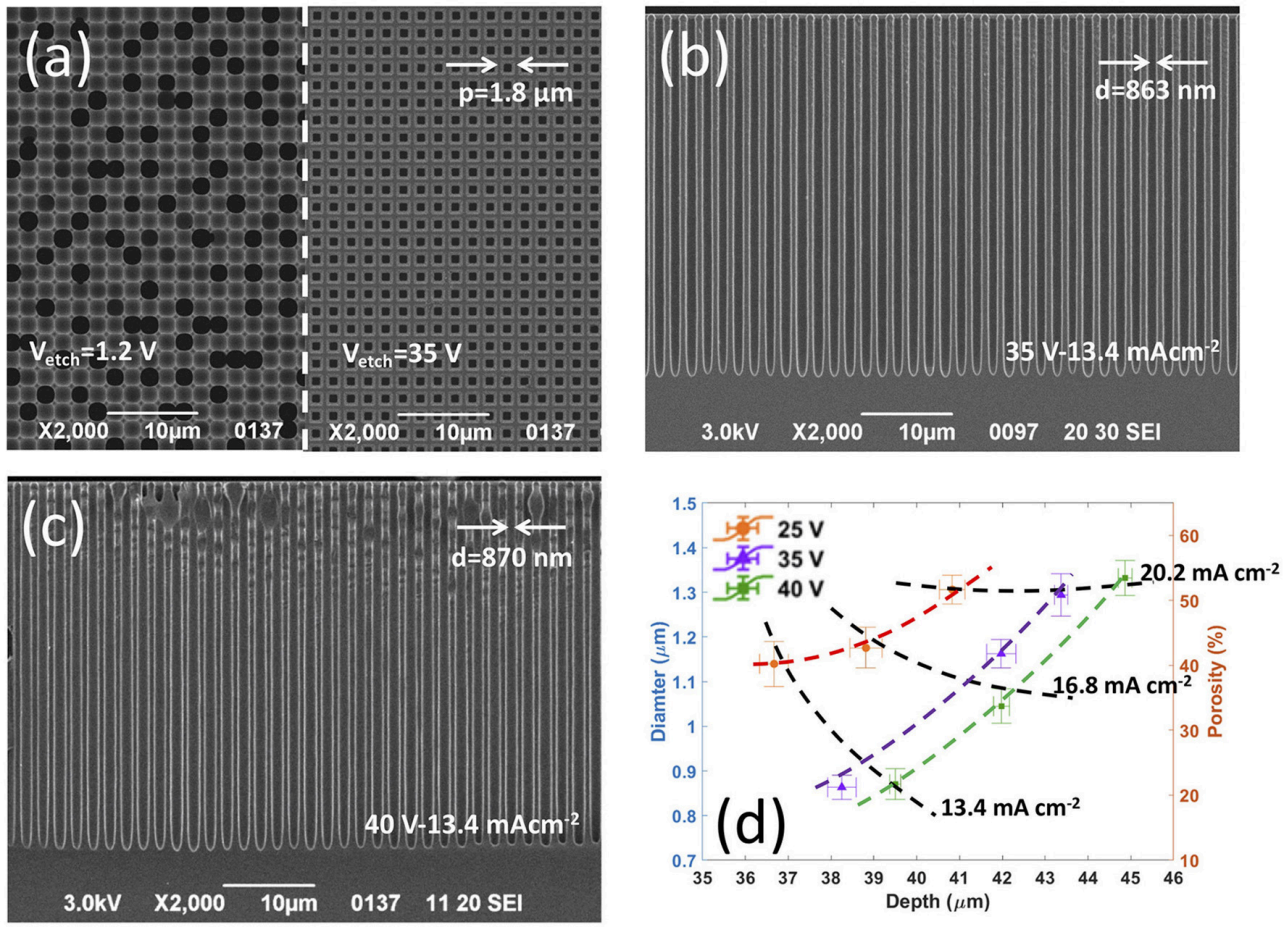
Influence of etching voltage *V*_*etch*_ and etching current density *J*_*etch*0_ value on the controlled etching of pores with sub-micrometric diameter and spacing of 1.8 μm. **(a)** Top-view SEM images showing (left side) the uncontrolled etching resulting by performing the BIEE with *V*_*etch*_ = 1.2 V and *J*_*etch*0_ = 13.4 mAcm^−2^, and (right side) a uniform array of sub-micrometric ordered pores featuring same diameter and depth, successfully achieved increasing the *V*_*etch*_ value to 35 V; **(b,c)** cross section SEM images showing uniform arrays of sub-micrometric ordered pores featuring same diameter and depth successfully obtained by BIEE performed with **(b)** *V*_*etch*_ = 35 V and *J*_*etch*0_ = 13.4 mAcm^−2^, and **(c)** *V*_*etch*_ = 40 V and *J*_*etch*0_ = 13.4 mAcm^−2^. **(d)** Morphological investigation of fully-uniform arrays of ordered pores etched at high anodic voltages, in terms of pore diameter, porosity, and depth: average values and standard deviations (error bars) of pore diameter (left axis) with corresponding experimental porosity (right axis) as a function of pore depth (average values and standard deviations) for each of the *V*_*etch*_ and *J*_*etch*0_ values investigated.

Figure 1a (left side) shows a typical top-view SEM image of an array of pores prepared by anodic etching under back-side illumination at *V*_*etch*_ = 1.2 V and *P*_*e*_ = 17.2% (*J*_*etch*0_ = 13.4 mA cm^−2^) of low-doped *n*-type silicon pre-patterned with regular lattices of square holes with size of 1 μm and pitch of 1.8 μm. The etching results in randomly-organized pores with non-constant spacing and average diameter of about 2 μm, which are uncorrelated from the pre-patterned layout, as confirmed by SEM analysis of the cross-section of the pore array (Figure S2a). Increasing the expected porosity value to 21.5 and 25.8% (etching current density value *J*_*etch*0_ to 16.8 and 20.2 mA cm^−2^, respectively), while keeping the *V*_*etch*_ value unchanged (1.2 V), does not significantly improve the etching outcome (Figures S2b,c). This agrees with the state-of-the-art literature on the BIEE of pores in low-doped silicon at low anodic voltage (Barillaro and Strambini, 2010), according to which both minimum diameter and spacing exist for the controlled dissolution of silicon in acidic electrolytes, which depend on silicon resistivity, below which the anodic etching of regular patterns of square holes cannot be controlled to achieve a regular array of pores. For instance, for *n*-type silicon electrodes with resistivity of a few Ω cm, typically used in microelectronics, minimum values for diameter and spacing of pores above which the anodic etching can be fully controlled are about 1 and 2 μm, respectively. Below these boundary values, competition for collection of holes, photogenerated on the silicon back-side, at the tips of adjacent pores is very high. In fact, after an initial nucleation phase, during which silicon dissolution proceeds with the formation of pits in correspondence of defect sites pre-patterned at the silicon surface, photogenerated hole collection is no longer uniform over time and space, so that some pits collect more holes than others within the array. The former keep growing and increase their diameter to reach a roughly constant steady-state value over depth; the latter stop growing after a given depth, which depends on the growth of neighbor pores (Figures S2a–c).

Intense electric-field establishing across the depletion region at the pore tips (defects, in the nucleation phase) is known to induce an effective hole-focusing at low *V*_*etch*_ values when diameter and spacing of pores (defects) is >2 μm. We argue that severe overlap of space charge regions of adjacent pores occurring when the pore spacing reduces below 2 μm leads to a defocusing of electric-field lines at the pore tips, which affects the collection of photogenerated holes and enhances surface non-homogeneities, giving rise to a non-uniform sharing of photogenerated holes and, in turn, to an uncontrolled etching/growth of the pores.

Experiments at higher anodic voltage values *V*_*etch*_, namely from 15 to 40 V, using the same current density values above reported for 1.2 V, were carried out to investigate the effect of an increasing electric-field on the focusing at the pore tips of holes photogenerated on the silicon back-side. Experimental results on the electrochemical etching of pores at increased anodic voltages are reported in Figures 1b,c and Figures S2d–t. Increasing the *V*_*etch*_ value turned out to have a beneficial effect on the controlled anodic etching of pre-patterned lattices of square holes with spacing < 2 μm. In fact, the number of dying pores significantly reduces as the etching voltage increases from 1.2 to 15 and 20 V, for any given etching current density value, and the correlation between etched/growing pores and surface pattern greatly improves (Figures S2d–i). Remarkably, for a given anodic voltage value, the number of dying pores reduces as the etching current density increases, then becomes zero at 20 V and 20.2 mA cm^−2^ (*P*_*e*_ = 25.8%), so enabling the fabrication of a regular array of pores with uniform depth and diameter and without missing pores (Figure S2i). A further increase of the *V*_*etch*_ value to 25, 35, and 40 V (Figures S2l–t) highlights that, as the anodic voltage increases the etching of pores can be fully controlled also at smaller *J*_*etch*0_ values, namely 13.4 (*P*_*theo*_ = 17.2%) and 16.8 mA cm^−2^ (*P*_*theo*_ = 21.5%). Notice that, at *V*_*etch*_ of 35 and 40 V, arrays of regular pores with no missing pores were successfully prepared with a submicrometric dimater of about about 860 nm at *J*_*etch*0_ = 13.4 mA cm^−2^ (Figures 1b,c).

### Effect of high anodic-voltage on geometrical features of regular arrays of high-density high-aspect-ratio pores etched in *n*-type silicon

A thorough morphological investigation of regular arrays of pores (with no missing pores) etched at different high anodic voltages/current densities was carried out to infer into the effect of etching parameters on pore diameter, porosity, and depth. Figure 1d shows experimental values (average value and standard deviation, sd) of pore diameter (*d*_*m*_, left axis) and array porosity (*P*_*m*_, right axis) vs. pore depth (*h*_*m*_), achieved at anodic voltages of 25, 35, and 40 V and current densities of 13.4, 16.8, and 20.2 mA cm^−2^. Coefficient of variation %CV (by definition, the ratio between standard deviation and mean values) values average 3.4 and 0.7% for diameter/porosity and depth, respectively, highlighting that the etched pores are uniform over the whole array both in the in-plane and out-of-plane directions. For a given anodic voltage, an increase of both pore diameter *d*_*m*_ and array porosity *P*_*m*_ occurs as the etching current density *J*_*etch*0_ increases. Remarkably, once etching current density and etching time are given, both diameter/porosity and depth of the pores strongly depend on the anodic voltage value. For instance, at *J*_*etch*0_ = 13.4 mA cm^−2^ (*t*_*etc*__h_ = 2,000 s) the anodic etching of the pre-patterned square hole lattice gives rise to a regular array of sub-micrometric pores with diameter of 870 nm (sd = 35 nm) and depth of 39.5 μm (sd = 131 nm) at 40 V, and diameter of 863 nm (sd = 27 nm) and depth of 38.3 μm (sd = 335 nm) at 35 V, whereas pores etched at 25 V feature a diameter of 1.14 μm (sd = 50 nm) and depth of 36.7 μm (sd = 331 nm).

This differs from what expected for pores prepared in silicon through electrochemical etching at anodic voltage values close to the electropolishing voltage peak (i.e., a few Volts). In this case, once both etching current density and etching time are chosen, the electrochemical etching leads to pores with same diameter/porosity and depth, regardless of the anodic voltage value, if diameter and spacing of the etched pores are above 1 and 2 μm, respectively. Figure S3a shows expected values for diameter, porosity, and depth (calculated using Equations 1, 2, 6, respectively) of pores etched at current densities of 13.4, 16.8, and 20.2 mA cm^−2^, regardless of the anodic voltage value. From the comparison between Figure 1d and Figure S3a, we can infer that, despite a properly controlled etching, a significant deviation of pore diameter and, in turn, array porosity from the expected values occurs at high anodic voltages for *J*_*etch*0_ values of 16.8 and 20.2 mA cm^−2^, regardless of the *V*_*etch*_ value. On the contrary, the arrays etched at the lowest *J*_*etch*0_ value (i.e., 13.4 mA cm^−2^) and highest *V*_*etch*_ values (i.e., 35 and 40 V) featured experimental diameter and porosity of about 870 nm and ~20%, respectively, in good agreement with expected values (Figure S3a). Moreover, the comparison between Figures 1d, S3a also highlights a marked mismatch between measured and expected pore depths, thus pointing out that the growth rate depends on both *J*_*etch*0_ and *V*_*etch*_ values when the etching is performed at high anodic voltages.

Experimental results on pore depth, diameter, and porosity were further used to calculate the total amount of silicon dissolved (Figure S3b), for which a good agreement with expected values was obtained (dashed line in Figure S3b) when the electrochemical etching was performed at the highest *J*_*etch*0_ value (i.e., 20.2 mA cm^−2^), regardless of the *V*_*etch*_ value. On the other hand, the mismatch between calculated and expected amount of silicon dissolved increased as the etching current density decreased, with a maximum mismatch obtained for fully uniform arrays of sub-micrometric pores etched at 13.4 mA cm^−2^ with 35 and 40 V. We argue that, such a mismatch can be ascribed to a variation of the silicon dissolution valence at high anodic voltages, when low etching current densities are employed.

### Physics of high anodic-voltage etching of high-density pores in *n*-type silicon electrodes

The back-side illumination electrochemical etching of low-doped *n*-type silicon electrodes involves the flow of photogenerated holes through the depletion region establishing within the silicon electrode, at the electrolyte/silicon interface. Therefore, a steady etching of pores within a silicon electrode pre-patterned with a regular lattice of defects requires that a steady flow of holes is established and sustained both in time and space through the space charge region at the pore tip. Both flow intensity and collection area of holes at the pore tip define, at a given etching time, the geometrical features of the resulting array of pores, namely diameter/porosity and length. Specifically, the flow intensity is mainly set by the electric-field establishing within the depletion region, while the collection area is mainly affected by the depletion region width establishing at the pore tip. Therefore, experimental results achieved on the electrochemical etching of high-density pores were tentatively interpreted in terms of the depletion region establishing at the pore tip at low and high anodic voltages.

**Figure 2:**
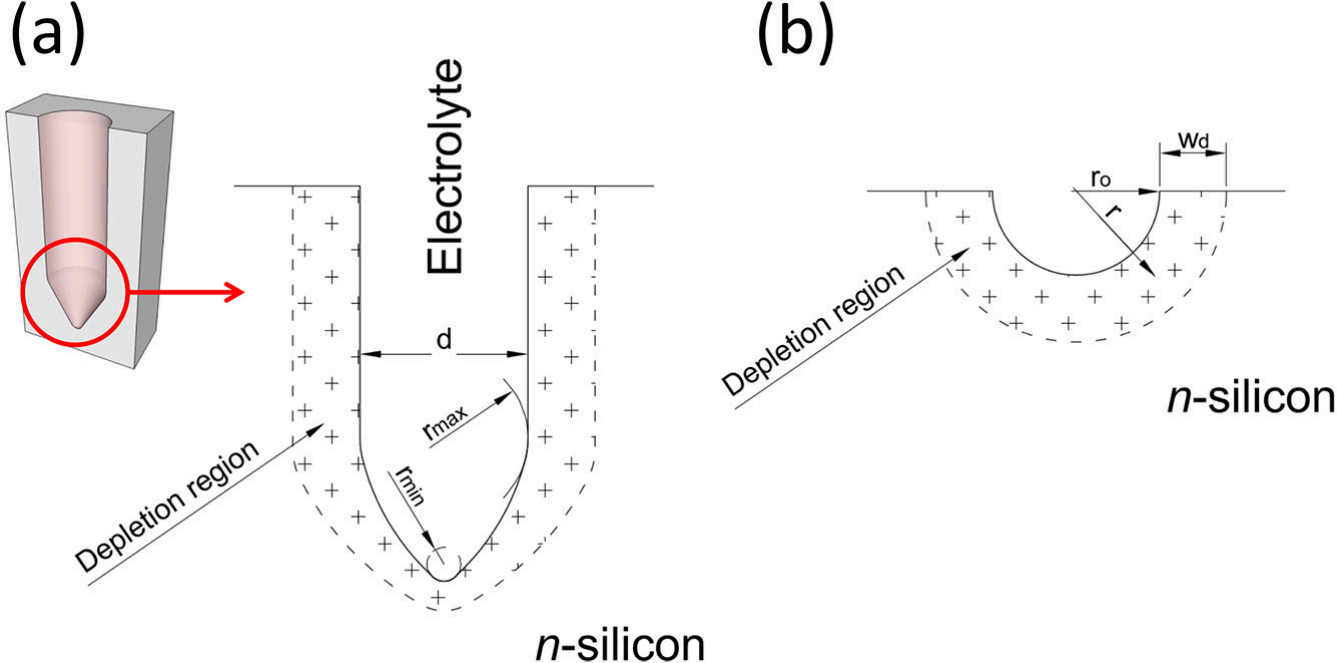
Sketch of the electrolyte/silicon interface of a pore, with depletion region establishing into the silicon substrate highlighted. **(a)** Pore-shaped electrolyte/silicon interface; **(b)** hemispherical-shaped electrolyte-silicon interface. The radius of curvature of the electrolyte/silicon interface at the pore tip raises monotonically from the tip apex, minimum value *r*_*min*_, to the pore radius, maximum value *r*_*max*_ = *d*/2, assuming for the pore a circular cross-section with a diameter *d*.

The radius of curvature of the electrolyte/silicon interface is one of the key parameters affecting the depletion region developing within the silicon electrode, both in terms of width of the space charge region and intensity of the electric-field inside it. From SEM cross-sections of pores fabricated at low and high anodic voltages (Figures S2a–t), it is apparent that the pore tips become more elongated as the anodic voltage increases, so that the curvature radius at the apex of the pore tips is smaller at higher anodic voltage, with respect to that a low anodic voltage. This is sketched in Figure 2a, which shows as the curvature radius of the pore tip, and, in turn, of the electrolyte/silicon interface, increases monotonically moving from the tip apex, minimum value *r*_*min*_, to the pore base, maximum value *r*_*max*_ = *d*/2, assuming for the pore a circular cross-section with a diameter *d*. A simplified theoretical model was implemented that takes variation of the curvature radius along the pore tip into account and allows the effect of anodic voltage on depletion region width and, in turn, electric-field intensity along the pore tip to be investigated. In the model, the tip surface is schematized with independent spherical surfaces/junctions with radius of curvature *r*_0_ variable between *r*_*min*_ and the tip apex and *r*_*max*_ at the pore base (Figure 2a). One single pore/tip is analyzed, neglecting possible contributions of neighbor pores to depletion region and, in turn, electric-field at the pore tip. Also, the anodic voltage *V*_*etc*__h_ is assumed to fully drop across the space charge region formed in the silicon electrode, neglecting possible voltage drops across the space charge region established in the electrolyte and along the resistive paths between voltage source and electrolyte/silicon interface.

For a spherical electrolyte/silicon interface with curvature radius *r*_0_ (Figure 2b), the following relations for electric-field *E*(*r*) and electric potential *V*(*r*) as a function of *r*_0_ can be obtained by solving the Poisson equation in the silicon electrode (Muller and Kamins, 1977):

(7) $$\begin{array}{r} E\left(r\right)=\frac{qN_{D}}{3\varepsilon_{s}}\left[-r+\frac{{\left(r_{0}+w_{d}\right)}^{3}}{r^{2}}\right] \end{array}$$

(8) $$\begin{array}{r} V\left(r\right)=\frac{qN_{D}}{6\varepsilon_{s}}\left[r^{2}+\frac{2{\left(r_{0}+w_{d}\right)}^{3}}{r}-3{\left(r_{0}+w_{d}\right)}^{2}\right] \end{array}$$

being *r* the distance from center of the spherical surface, ε the dielectric constant of silicon, *N*_*D*_ the ionized donor density, *q* the elementary electron charge, and *w*_*d*_ the width of the depletion region. Figure 2b shows a cross-section of a spherical electrolyte/silicon interface with curvature radius *r*_0_, also highlighting the depletion region *w*_*d*_ established within the silicon electrode.

**Figure 3:**
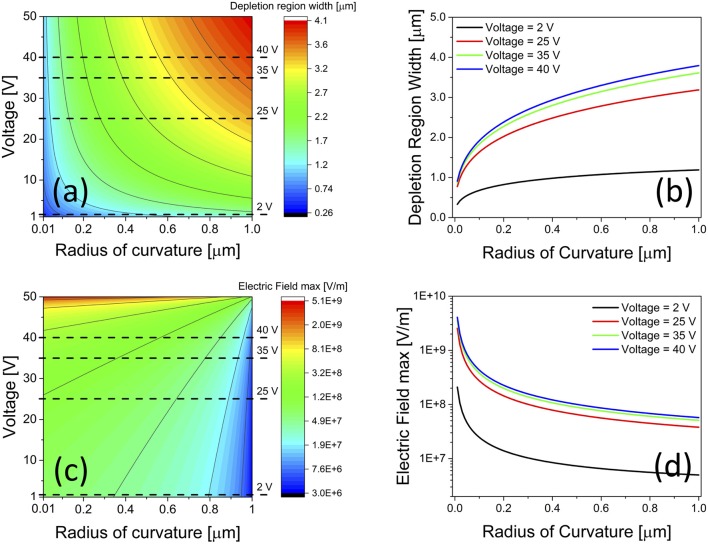
Theoretical results on depletion region width and maximum electric-field intensity at the electrolyte/silicon interface of a pore, as a function of both anodic voltage and curvature radius values. **(a,c)** Contour plot of depletion region width *w*_*d*_ and maximum intensity of electric-field *E*_*max*_ in the silicon substrate as a function of electrolyte/silicon interface anodic voltage and curvature radius; **(b,d)** depletion region width *w*_*d*_ and maximum intensity of electric-field *E*_*max*_ in the silicon substrate as a function of electrolyte/silicon interface curvature radius, for different anodic voltage values, namely 2, 25, 35, and 40 V, corresponding to dashed lines in **(a,b)**.

For a given voltage difference *V*_*d*_ applied between electrolyte and silicon, a relationship between depletion region width *w*_*d*_ and applied voltage *V*_*d*_ is obtained by assuming *V*(*r*_0_) = –*V*_*d*_ in Equation (8) (see Supplementary Material). This relationship was used to plot the depletion region width *w*_*d*_ in the silicon electrode as a function of the voltage *V*_*d*_ applied across the silicon/electrolyte interface and of the curvature radius *r*_0_ of the silicon/electrolyte interface (Figure 3a). Figure 3a shows a contour plot of the depletion region width *w*_*d*_ in the silicon electrode, as a function of the anodic voltage *V*_*d*_ (between 0 and 50 V) applied across the silicon/electrolyte interface and of the curvature radius *r*_0_ (between 100 and 1,000 nm) of the silicon/electrolyte interface. Figure 3b shows the relationship between depletion region width *w*_*d*_ and curvature radius *r*_0_ for increasing values of *V*_*d*_ (corresponding to the dashed lines in Figure 3a).

By replacing *w*_*d*_ into Equation (7) with its expression derived above, a relationship between the maximum electric-field *E*_*max*_ = *E*(*r*_0_) establishing within the depletion region in the silicon electrode and the voltage *V*_*d*_ applied is obtained (see Supplementary Material). This was used to plot the maximum value of the electric-field *E*_*max*_ = *E*(*r*_0_) within the depletion region in the silicon electrode as a function of the anodic voltage *V*_*d*_ applied across the silicon/electrolyte interface and of the curvature radius *r*_0_ of the silicon/electrolyte interface (Figure 3c). Figure 3c shows a contour plot of the maximum value of the electric-field *E*_*max*_ = *E*(*r*_0_) establishing within the depletion region in the silicon electrode, as a function of the anodic voltage *V*_*d*_ (between 0 and 50 V) applied across the silicon/electrolyte interface and of the curvature radius *r*_0_ (between 100 and 1,000 nm) of the silicon/electrolyte interface. Figure 3d shows the relationship between maximum electric-field *E*_*max*_ and curvature radius *r*_0_ for increasing values of the applied voltage *V*_*d*_ (corresponding to dashed lines in Figure 3d). As expected, an increase of both depletion region width *w*_*d*_ and electric-field intensity *E*_*max*_ at the pore tip occurs as the anodic voltage *V*_*d*_ is augmented, regardless of the curvature radius at the tip. However, the degree by which both depletion region width *w*_*d*_ and electric-field intensity *E*_*max*_ are augmented, depends on the curvature radius value. For instance, as the curvature radius at the tip reduces from 500 nm (i.e., pore base) to 100 nm (i.e., pore apex), the ratio between width of the depletion region at the pore base and tip apex increases from 3.1 to 3.4 (Figure S4a), and the ratio between maximum electric-field intensity at the tip apex and pore base increases from 27 to 42 (Figure S4b), as the anodic voltage is augmented from 2 to 40 V.

We argue that, for array of pores with spacing much larger than twice the depletion region, the pores of the array can be considered isolated from each other, so that low values (e.g., 2 V) of the anodic voltage *V*_*etch*_ are enough to sustain an effective hole focusing at the pore tip apex, where the curvature radius of the electrolyte/silicon interface is smaller than that at the pore base, thanks to the simultaneous lower value of *w*_*d*_ and higher value of *E*_*max*_ at the tip apex with respect to the pore base. This allows all pores of the arrays to develop and grow steadily. On the other hand, for array of pores with spacing of the same order of the depletion region or smaller (i.e., high-density array of pores), the pores in the array cannot be considered isolated anymore. In this case, the overlap of the depletion region between adjacent pores sensibly reduces the differences between curvature radius at the tip apex and pore base and, in turn, hole focusing efficiency at the tip apex. Tip apex and pore base now share photogenerated holes, thus producing morphologically-driven instabilities in the electrochemical etching of pores, for low anodic voltage values. Conversely, the use of high values (e.g., >20 V) for the anodic voltage *V*_*etch*_ allows the ratios between depletion region widths *w*_*d*_ at the tip apex and pore base (Figure S4a) and between maximum electric-field intensities *E*_*max*_ at the tip apex and pore base (Figure S4b) to be further enhanced with respect to those at low anodic voltage. This enables an efficient electric-field driven focusing of holes at the tip apex also for high-density array of pores with spacing smaller than the depletion region width, and, in turn, a stable pore growth/etching, in agreement with experimental results obtained at high anodic voltage in this work. Indeed, for a given etching current density value, whereas randomly-distributed pores were obtained from low-doped silicon substrates pre-patterned with a lattice of holes with spacing of 1.8 μm using a low anodic voltage (1.2 V), perfect arrays of pores were achieved at high anodic voltages (>20 V) using the same pre-patterned silicon substrates, thanks to the enhanced electric-field focusing of holes at the tip apex of the pores, with respect to the pore base, which allowed the etching of pore tips to be preferentially initiated and steadily sustained.

## Conclusions

In conclusion, the controlled fabrication of high-density (~30 × 10^6^ cm^−2^) regular arrays of pores featuring high-depth (up to ~45 μm), high-aspect-ratio (from ~35 to ~45), and spacing of 1.8 μm was successfully achieved by back-side illumination electrochemical etching at high anodic voltage (from 20 to 40 V) of low-doped (resistivity 3–8 Ω cm) *n*-type silicon using low-HF-concentration etchants (5% by vol. in deionized water). Regular arrays of sub-micrometric pores featuring a diameter of 863 nm (sd = 27 nm) and 870 nm (sd = 35 nm), were successfully etched at 35 and 40 V, respectively, with *J*_*etch*0_ = 13.4 mA cm^−2^. A theoretical model was proposed, which allows experimental results to be interpreted in terms of an electric-field enhanced focusing of holes at the tip apex of the pores, with respect to the pore base, at high anodic voltages, which enables the etching of the pore tips to be preferentially initiated and steadily sustained over time and space.

The controlled anodic etching of submicrometer pores in low-doped *n*-type silicon envisages the possibility to scale the electrochemical micromachining (ECM) technology down to the mesoscale, through a better understanding of the silicon dissolution at high anodic biasing and by further optimization of the etching conditions, in terms of composition of the etching solution. This would open noteworthy applications in the fields of (though not limited to) nanomedicine, nanoelectromechanical systems (NEMS), and nanoelectronics.

## Author contributions

CC and GP carried out the experiments. LS designed the theoretical model. GB conceived the idea and supervised the research. All authors analyzed and discussed the results and wrote the manuscript.

### Conflict of interest statement

The authors declare that the research was conducted in the absence of any commercial or financial relationships that could be construed as a potential conflict of interest.

## References

[B1] Barillaro G. (2015). Silicon electrochemical micromachining technology: the good, the bad, and the future. ECS Trans. 69, 39–46. 10.1149/06902.0039ecst

[B2] Barillaro G. Diligenti A. Nannini A. Strambini L. M. (2005). Gas sensors based on silicon devices with a porous layer. Phys. Stat. Sol. C 2, 3424–3428. 10.1002/pssc.200461202

[B3] Barillaro G. Nannini A. Pieri F. (2002b). Dimensional constraints on high aspect-ratio silicon microstructures fabricated by HF photoelectrochemical etching. J. Electrochem. Soc. 149, C180–C185. 10.1149/1.1449953

[B4] Barillaro G. Nannini A. Piotto M. (2002a). Electrochemical etching in HF solution for silicon micromachining. Sens. Actuators A 102:195–201. 10.1016/S0924-4247(02)00385-0

[B5] Barillaro G. Pieri F. (2005). A self-consistent theoretical model for macropore growth in *n*-type silicon. J. Appl. Phys. 97:116105 10.1063/1.1915534

[B6] Barillaro G. Strambini L. M. (2010). Controlling macropore formation in patterned *n*-type silicon: existence of a pitch-dependent etching current density lower bound. Electrochem. Commun. 12, 1314–1317. 10.1016/j.elecom.2010.07.008

[B7] Bassu M. Surdo S. Strambini L. M. Barillaro G. (2012). Electrochemical micromachining as an enabling technology for advanced silicon microstructuring. Adv. Funct. Mater. 22, 1222–1228. 10.1002/adfm.201102124

[B8] Cozzi C. Polito G. Kolasinski K. Barillaro G. (2017). Controlled microfabrication of high-aspect-ratio structures in silicon at the highest etching rates: the role of H_2_O_2_ in the anodic dissolution of silicon in acidic electrolytes. Adv. Funct. Mater. 7:1604310 10.1002/adfm.201604310

[B9] Cozzi C. Polito G. Strambini L. M. Barillaro G. (2015). Electrochemical preparation of in-silicon hierarchical networks of regular out-of-plane macropores interconnected by secondary in-plane pores through controlled inhibition of breakdown effects. Electrochim. Acta 187, 552–559. 10.1016/j.electacta.2015.11.006

[B10] Delalat B. Cozzi C. Ghaemi S. R. Polito G. Kriel F. E. Michl T. D. (2018). Microengineered bioartificial liver chip for drug toxicity screening. Adv. Funct. Mater. 28:1801825 10.1002/adfm.201801825

[B11] Föll H. Christophersen M. Carstensen J. Hasse G. (2002). Formation and application of porous silicon. Mat. Sci. Eng. R. 39, 93–141. 10.1016/S0927-796X(02)00090-6

[B12] Harding F. J. Surdo S. Delalat B. Cozzi C. Elnathan R. Gronthos S.. (2016). Ordered silicon pillar arrays prepared by electrochemical micromachining: substrates for high-efficiency cell transfection. ACS Appl. Mater Interface 8, 29197–29202. 10.1021/acsami.6b07850 27744675

[B13] Kemell M. Ritala M. Leskelä M. Ossei-Wusu E. Carstensen J. Föll H. (2007). Si/Al_2_O_3_/ZnO:Al capacitor arrays formed in electrochemically etched porous Si by atomic layer deposition. Microelectron. Eng. 84, 313–318. 10.1016/j.mee.2006.10.085

[B14] Lehmann V. (1993). The physics of macropore formation in low doped *n*-type silicon. J. Electrochem. Soc. 140, 2836–2843. 10.1149/1.2220919

[B15] Lehmann V. Föll H. (1990). Formation mechanism and properties of electrochemically etched trenches in *n-type* silicon. J. Electrochem. Soc. 137, 653–659. 10.1149/1.2086525

[B16] Matthias S. Müller F. Schilling J. Gösele U. (2005). Pushing the limits of macroporous silicon etching. Appl. Phys. A 80, 1391–1396. 10.1007/s00339-004-3193-x

[B17] Muller R. S. Kamins T. I. (1977). Device Electronics for Integrated Circuits. New York, NY: John Wiley & Sons.

[B18] Polito G. Surdo S. Robbiano V. Tregnano G. Cacialli F. Barillaro G. (2013). Two-dimensional array of photoluminescent light-sources by selective integration of conjugated luminescent polymers into three-dimensional silicon microstructures. Adv. Optical Mater. 1, 894–898. 10.1002/adom.201300288

[B19] Rönnebeck S. Carstensen J. Ottow S. Föll H. (1999). Crystal orientation dependence of macropore growth in *n*-type silicon. Electrochem. Solid State Lett. 2, 126–128. 10.1149/1.1390756

[B20] Surdo S. Carpignano F. Silva G. Merlo S. Barillaro G. (2013). An all-silicon optical platform based on linear array of vertical high-aspect-ratio silicon/air photonic crystals. Appl. Phys. Lett. 103:171103 10.1063/1.4826146

[B21] Surdo S. Carpignano F. Strambini L. M. Merlo S. Barillaro G. (2014). Capillarity-driven (Self-Powered) one-dimensional photonic crystals for refractometry and (Bio)sensing. RSC Adv. 4, 51935–51914. 10.1039/C4RA09056J

[B22] Surdo S. Merlo S. Carpignano F. Strambini L. M. Trono C. Giannetti A.. (2012). Optofluidic microsystems with integrated vertical one-dimensional photonic crystals for chemical analysis. Lab Chip 12, 4403–4415. 10.1039/c2lc40613f 22930245

[B23] Uhlir A. (1956). Electrolytic shaping of germanium and silicon. Bell Syst. Tech. J. 35, 333–347. 10.1002/j.1538-7305.1956.tb02385.x

