# Supplementary material for: High Anodic-Voltage Focusing of Charge Carriers in Silicon Enables the Etching of Regularly-Arranged Submicrometer Pores at High Density and High Aspect-Ratio
Source: Front Chem. 2018 Nov 30;6:582. doi: 10.3389/fchem.2018.00582 (PMC6287200; doi:10.3389/fchem.2018.00582)
Supplement: Supplementary file 1 [file Data_Sheet_1.pdf]

Supplementary Material

High Anodic-Voltage Focusing of Charge Carriers in Silicon Enables the Etching of Regularly-Arranged Submicrometer Pores at High Density and High Aspect-Ratio

Chiara Cozzi[1†], Giovanni Polito[1†], Lucanos M. Strambini[2], Giuseppe Barillaro[1,2*]

[1]Dipartimento di Ingegneria dell'Informazione, Università di Pisa, via G. Caruso 16, 56122 Pisa, Italy

[2]Istituto di Elettronica e di Ingegneria dell'Informazione e delle Telecomunicazioni, Consiglio Nazionale delle Ricerche, via G. Caruso 16, 56122, Pisa, Italy

[†]These authors contributed equally to this work.

*** Correspondence:** Corresponding Author: g.barillaro@iet.unipi.it

1 Supplementary Data

1.1 Electrochemical etching setup

The electrochemical etching setup includes a three-electrode polytetrafluorethylene (PTFE) electrochemical etch-cell with a volume of 400 cm^3. The front-side of the silicon electrode is in contact with the electrolyte through a circular-shaped aperture identifying an etching area (E_A) of ~0.64 cm^2. A halogen lamp (250 W) positioned 5 cm away from the silicon electrode is used to photogenerate a hole current by illuminating the silicon back-side through a circular window (1.3 cm^2) in the metal electrode providing the electrical contact to the sample itself. The photogenerated hole current was properly controlled as the etching progressed changing the lamp power and, in turn, the illumination intensity by means of a feedback loop performed using a proportional-integral-derivative (PID) controller (Eurotherm 2604). An IR filter with a cut-off wavelength of 750 nm was placed between the lamp and the silicon sample to avoid charge carrier generation close to the silicon surface in contact with the electrolyte, whereas the electrolyte was stirred during the etching to enhance the etching uniformity. The working temperature was kept constant at 22 °C for the entire etching time through a second feedback loop implemented using another PID controller. A platinum disk placed at 1 cm from the silicon anode and a platinum wire placed at about 2 mm from the silicon electrode were used as cathode and pseudo-reference electrodes immersed in the HF-aqueous electrolyte, respectively. A Source Measure Unit (Keithley 2410 Source Meter) was used to apply the anodization voltage between the silicon slab and the reference electrode, and to monitor the photogenerated current.

1.2 Electrolyte/silicon interface radius of curvature and anodic voltage effects on the the depletion region establishing at the pore tip

Assuming that the pore tip has a spherical surface with curvature radius r_0 varying from r_{min} at the apex to r_{max} at the pore base, it is feasible to solve the Poisson equation at the electrolyte/silicon interface:

$$\frac{1}{r^2}\frac{d}{dr}\left[r^2E(r)\right] = \frac{\rho(r)}{\varepsilon} \qquad \text{(S1)}$$

being r the distance from center of the spherical surface, ε the dielectric constant of silicon, $E(r)$ the electric field, and $\rho(r)$ the charge density. Figure 2b shows a cross-section of the pore tip spherical surface with curvature radius r_0, also highlighting the depletion region w_d established within the silicon electrode. The charge density $\rho(r)$ into the silicon volume is $-qN_D$ for $r_0 \leq r \leq r_0 + w_d$ and zero elsewhere, being N_D the ionized donor density, q the elementary electron charge, and w_d the width of the depletion region. By solving equation S1 for silicon using fully-depletion approximation (i.e. boundary conditions for electric field $E(r_0 + w_d) = 0$ V/m and potential $V(r_0 + w_d) = 0$ V (Muller and Kamins, 1977)), the following relationships between $E(r)$, $V(r)$, and r_0 are obtained:

$$E(r) = \frac{qN_D}{3\varepsilon_S}\left[-r + \frac{(r_0+w_d)^3}{r^2}\right] \qquad \text{(S2)}$$

$$V(r) = \frac{qN_D}{6\varepsilon_S}\left[r^2 + \frac{2(r_0+w_d)^3}{r} - 3(r_0 + w_d)^2\right] \qquad \text{(S3)}$$

being $\varepsilon_s = 1.05365\text{x}10^{-10}$ F/m the silicon dielectric constant. For a given potential difference V_d applied across the electrolyte/silicon interface, it is possible to obtain a relationship between the depletion region width w_d within the silicon electrode and V_d by assuming $V(r_0) = -V_d$ in the equation S3:

$$V(r_0) = \frac{qN_D}{6\varepsilon_S}\left[{r_0}^2 + \frac{2(r_0+w_d)^3}{r_0} - 3(r_0 + w_d)^2\right] = -V_d \qquad \text{(S4)}$$

$$w_d^3 + \frac{3}{2}r_0w_d^2 + \frac{6\varepsilon_SV_d}{qN_D} = 0 \qquad \text{(S5)}$$

The width w_d of the depletion region into the silicon electrode is the solution of equation S5, given by solving a third order equation with real numbers coefficients as follow:

$$w_d^3 + aw_d^2 + bw_d + c = 0 \qquad \text{(S6)}$$

Being:

$$a = \frac{3}{2}r_0\,, \quad b = 0\,, \quad c = \frac{6\varepsilon_SV_d}{qN_D}$$

Assuming:

$$P = -\frac{a^2}{9} = -\frac{r_0^2}{4}\,, \quad Q = \frac{-2a^3+9ab-27c}{54} = -\frac{1}{8}\left(r_0^3 + 12\frac{\varepsilon_SV_d}{qN_D}r_0\right)$$

$$D = P^3 + Q^2 = \frac{3}{16}\frac{\varepsilon_S V_d}{qN_D} r_0^2 \left(2r_0^2 + 4\frac{\varepsilon_S V_d}{qN_D}\right)$$

Being D a positive number, equation S6 has only one real solution that can be figure out as follow:

$$R = \sqrt[3]{Q + \sqrt{D}}, \quad S = \sqrt[3]{Q - \sqrt{D}}$$

$$w_d = -\frac{a}{3} + R + S = -\frac{r_0}{2} + R + S \tag{S7}$$

Expression S7 relates the width w_d of the depletion region into the silicon substrate to the potential difference V_d and to the radius of curvature r_0 for a spherical shaped electrolyte/silicon interface. An increase of the electrolyte/silicon interface radius of curvature r_0 and/or the potential difference V_d leads to an increase of the depletion region width w_d. For these reasons, the depletion region width for the actual pore tip reaches a minimum at the apex. An increase of the electrolyte/silicon interface radius of curvature r_0 leads to a decrease of the maximum value assumed by the electric field into the silicon substrate. with a significant reduction of E_{max} only in the first 100 nm (of about one order of magnitude for r_0 moving from 10 nm to 100 nm). On the contrary, an increase of the potential difference V_d leads to an increase of E_{max}, with a variation of about one order of magnitude moving from the low-voltage to the high-voltage anodic regime. As shown by the simplified model implemented, the actual pore tip is the electrolyte/silicon interface region where the electric field is highest, and its value can be noteworthy increased by increasing the anodic voltage.

2 Supplementary Figures and Tables

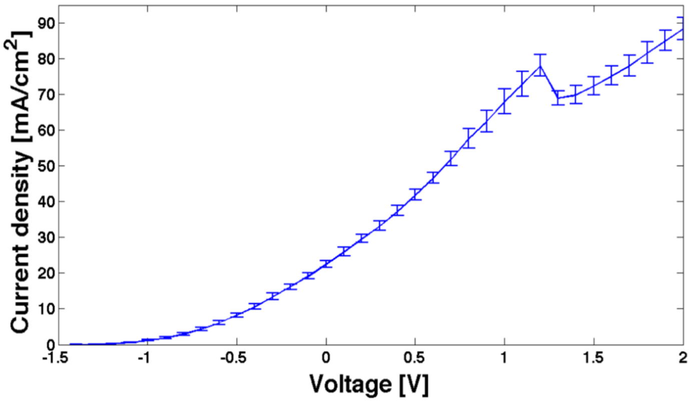

Figure S1. Electrochemical characterization of silicon dissolution in HF-based electrolyte solution by linear sweep voltammetry. Experimental current density-voltage curve (average value and standard deviation) of n-type silicon electrode in the presence of HF concentration of 5 vol% under back-side higher-intensity illumination (100% of the 250 W halogen lamp power).

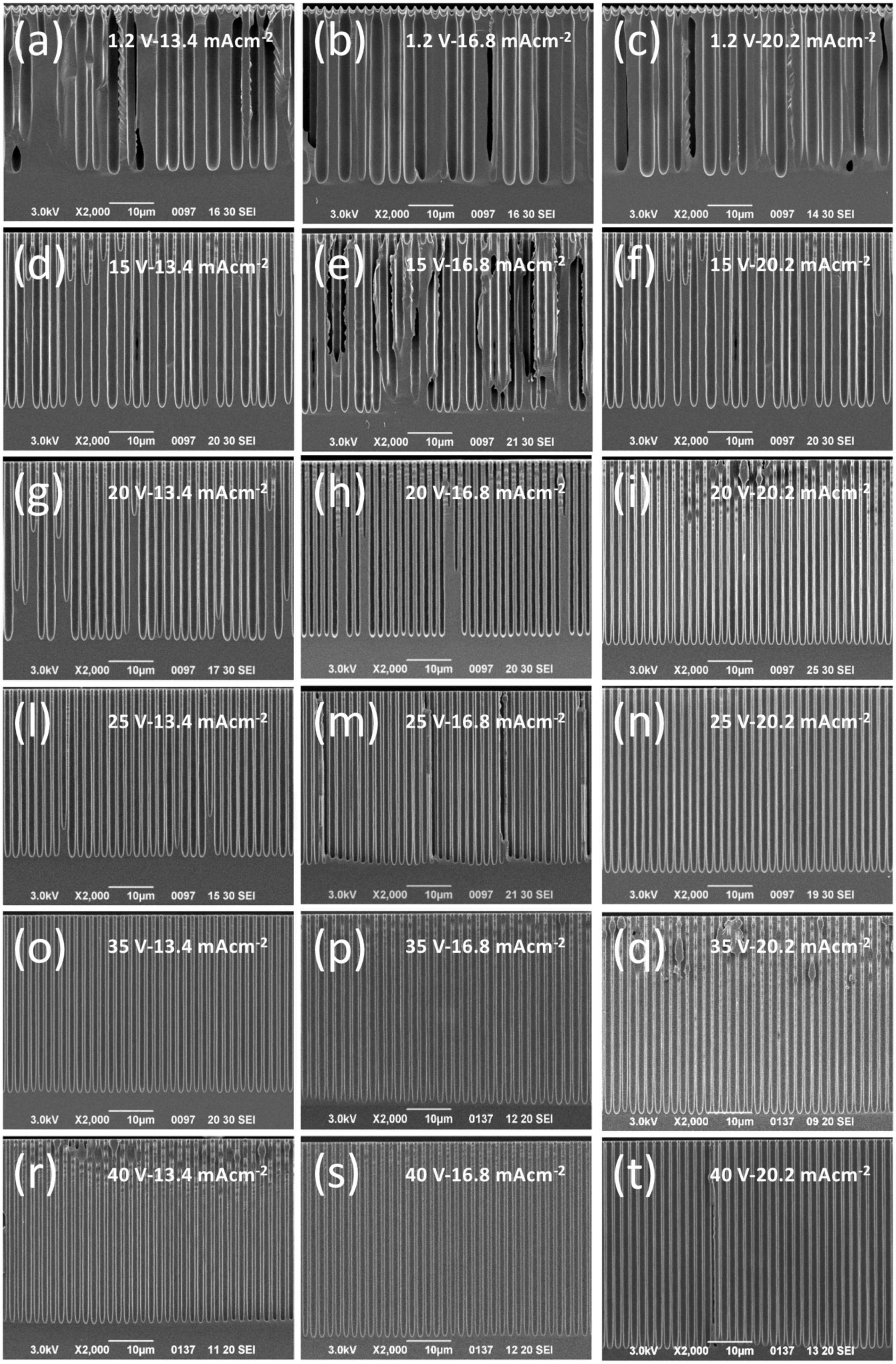
(a)
1.2 V-13.4 mAcm-2
3.0kV X2,000 10μm 0097 16 30 SEI
(b)
1.2 V-16.8 mAcm-2
3.0kV X2,000 10μm 0097 16 30 SEI
(c)
1.2 V-20.2 mAcm-2
3.0kV X2,000 10μm 0097 14 30 SEI
(d)
15 V-13.4 mAcm-2
3.0kV X2,000 10μm 0097 20 30 SEI
(e)
15 V-16.8 mAcm-2
3.0kV X2,000 10μm 0097 21 30 SEI
(f)
15 V-20.2 mAcm-2
3.0kV X2,000 10μm 0097 20 30 SEI
(g)
20 V-13.4 mAcm-2
3.0kV X2,000 10μm 0097 17 30 SEI
(h)
20 V-16.8 mAcm-2
3.0kV X2,000 10μm 0097 20 30 SEI
(i)
20 V-20.2 mAcm-2
3.0kV X2,000 10μm 0097 25 30 SEI
(l)
25 V-13.4 mAcm-2
3.0kV X2,000 10μm 0097 15 30 SEI
(m)
25 V-16.8 mAcm-2
3.0kV X2,000 10μm 0097 21 30 SEI
(n)
25 V-20.2 mAcm-2
3.0kV X2,000 10μm 0097 19 30 SEI
(o)
35 V-13.4 mAcm-2
3.0kV X2,000 10μm 0097 20 30 SEI
(p)
35 V-16.8 mAcm-2
3.0kV X2,000 10μm 0137 12 20 SEI
(q)
35 V-20.2 mAcm-2
3.0kV X2,000 10μm 0137 09 20 SEI
(r)
40 V-13.4 mAcm-2
3.0kV X2,000 10μm 0137 11 20 SEI
(s)
40 V-16.8 mAcm-2
3.0kV X2,000 10μm 0137 12 20 SEI
(t)
40 V-20.2 mAcm-2
3.0kV X2,000 10μm 0137 13 20 SEI

Figure S2. Influence of the etching voltage V_{etch} and the etching current density J_{etch0} value on the controlled etching of pores with sub-micrometric diameter and spacing below 2 µm. Cross section SEM images showing (a-c) the uncontrolled etching resulting by performing the BIEE with V_{etch}=1.2 V and (a) J_{etch0}=13.4 mA cm^{-2}, (b) J_{etch0}=16.8 mA cm^{-2}, and (c) J_{etch0}=20.2 mA cm^{-2}; (d-i) the uniform pore nucleation resulting from the beneficial effect of the increased etching voltage to (d-f) V_{etch}=15 V, and (g-i) with V_{etch}=20 V on the controlled etching with (d,g) J_{etch0}=13.4 mA cm^{-2}, (e,h) J_{etch0}=16.8 mA cm^{-2}, and (f,i) J_{etch0}=20.2 mA cm^{-2} leading to pore formation in all available defect sites in silicon samples etched by BIEE, showing only a limited number of shorter pores; (l-t) the uniform array of ordered pores featuring same depth and diameter successfully obtained by BIEE performed at (l-n) V_{etch}=25 V, (o-q) V_{etch}=35 V, and (r-t) with V_{etch}=40 V with (l,o,r) J_{etch0}=13.4 mA cm^{-2}, (m,p,s) J_{etch0}=16.8 mA cm^{-2}, and (n,q,t) J_{etch0}=20.2 mA cm^{-2}.

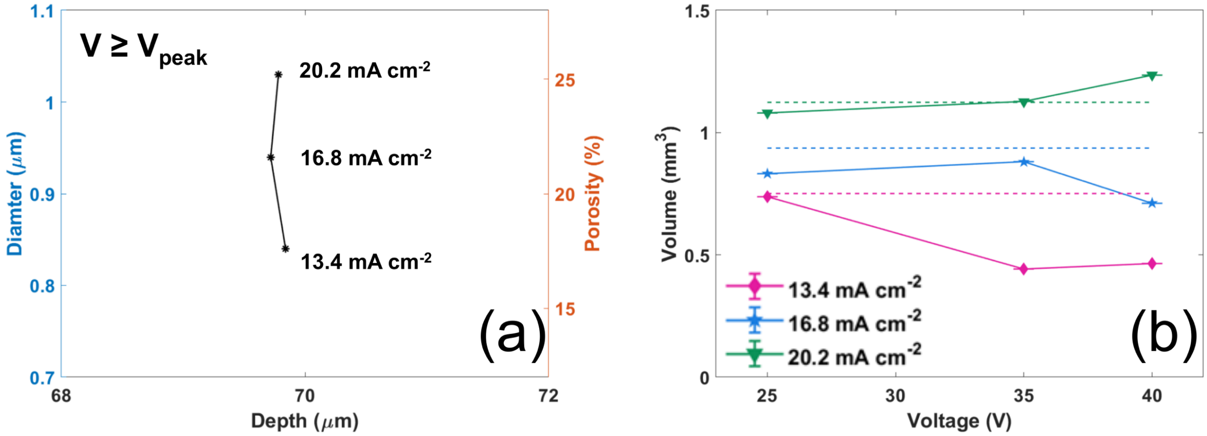

Figure S3. (a) Expected pore diameters (left axis) with corresponding expected porosities (right axis) as a function of expected pore depths corresponding to each of the J_{etch0} values investigated. (b) Calculated (solid lines) and theoretical (dashed lines) volume of dissolved silicon as a function of anodic voltages for each of the J_{etch0} values investigated.

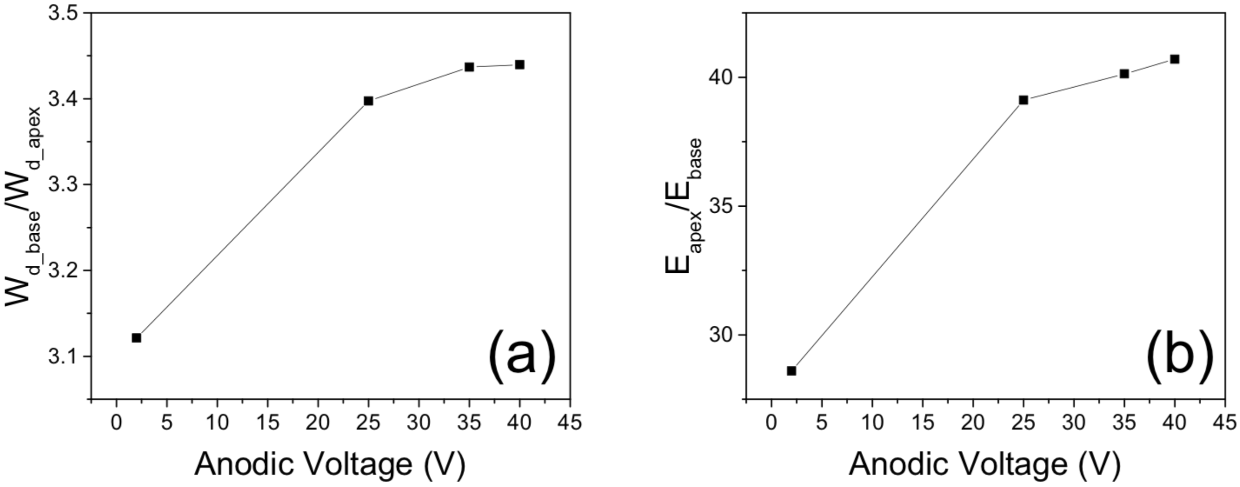

Figure S4. (a) Theoretical ratio between width of the depletion region at the pore base (i.e. curvature radius 500 nm) and tip apex (i.e. curvature radius 100 nm); (b) Theoretical ratio between maximum electric field intensity at the tip apex (i.e. curvature radius 100 nm) and pore base (i.e. curvature radius 500 nm).

**Anodization Voltage**	**Etching Current Density**		**Expected Porosity**	**Expected Diameter**
V_{etch} [V]	Starting value J_{etch0} [mA cm^{-2}]	Slope α [μA $s^{-1}cm^{2}$]	P_e [%]	d_e [μm]
1.2, 15, 20, 25, 35, 40	13.4	-0.938	17.2	0.842
	16.8	-1.219	21.5	0.942
	20.2	-1.453	25.8	1.032

Table S1: Parameters (i.e. anodic voltage and etching current density) used in this work for the electrochemical etching of silicon substrates with resistivity of 3-8 Ω cm pre-patterned with lattice of square holes with side of 1 μm and spacing of 1.8 μm, and values expected (i.e. porosity and diameter of pores).
